# Event Related Potentials Reveal Early Phonological and Orthographic Processing of Single Letters in Letter-Detection and Letter-Rhyme Paradigms

**DOI:** 10.3389/fnhum.2016.00176

**Published:** 2016-04-22

**Authors:** Sewon A. Bann, Anthony T. Herdman

**Affiliations:** School of Audiology and Speech Sciences, University of British ColumbiaVancouver, BC, Canada

**Keywords:** EEG, event related potentials, evoked potentials, global field power, language, letter processing

## Abstract

**Introduction**: When and where phonological processing occurs in the brain is still under some debate. Most paired-rhyme and phonological priming studies used word stimuli, which involve complex neural networks for word recognition and semantics. This study investigates early (<300 ms) and late (>300 ms) orthographic and phonological processing of letters.

**Methods**: Fifteen participants aged 20–35 engaged in three two-forced choice experiments, one letter-detection (LetterID) and two letter-rhyme (Paired-Rhyme and Letter-Rhyme) tasks. From the EEG recordings, event related potential (ERP) differences within and across task stimuli were found. We also calculated the global field power (GFP) for each participant. Accuracies and reaction times were also measured from their button presses for each task.

**Results**: Behavioral: Reaction times were 18 ms faster to letter than pseudoletter stimuli, and 27 ms faster to rhyme than nonrhyme stimuli. ERP/GFP: In the LetterID task, grand-mean evoked potentials (EPs) showed typical P1, N1, P2, and P3 waveform morphologies to letter and pseudoletter stimuli, with GFPs to pseudoletters being greater than letters from 160–600 ms. Across both rhyme tasks, there were greater negativities for nonrhyme than for rhyme stimuli at 145 ms and 426 ms. The P2 effect for rhyme stimuli was smaller than letter stimuli when compared across tasks.

**Conclusion**: Differences in early processing of letters vs. pseudoletters between 130–190 ms suggest that letters are processed earlier and perhaps faster in the brain than pseudoletters. The P2 effect between letter and rhyme stimuli likely reflect sublexical phonological processing. Together, findings from our study fill in evidence for the temporal dynamics of orthographic and phonological processing of single letters.

## Introduction

Phonological retrieval is a requisite process for reading acquisition by typically-developing readers (Wagner et al., 1993). When this occurs within the brain is still under some debate (Fiez and Petersen, 1998). The timing of phonological retrieval has been investigated using EEG and MEG (Kramer and Donchin, 1987; Rugg and Barrett, 1987; Coch et al., 2008b). Most of these studies used a paired-stimulus rhyming paradigm and showed that differences in responses to rhyme and nonrhyme stimuli occurred between 400–600 ms. From a neural dynamics perspective, this appears to be an extended delay for retrieving phonology of written text as compared to priming studies that showed phonological priming occurs within 150–200 ms (Holcomb and Grainger, 2006). In addition, most paired-rhyme and phonological priming studies used word stimuli, which can recruit complex neural processing/networks involved in word recognition and semantics. A few studies have used simple single-letter stimuli to help reduce recruitment of more complex processes (Taylor, 1993; Coch et al., 2008b). They also discovered event related potential (ERP) differences between rhyme and nonrhyme pairs of single letters to occur between 320–550 ms, encompassing the N450 rhyming effects (Coch et al., 2008b). Researchers have suggested that this effect is mainly due to phonological processing (Rugg and Barrett, 1987; Bentin et al., 1999; Penolazzi et al., 2006; Coch et al., 2008a). However, models of reading indicate that this is a fairly late interval for the initial stages of phonological processing to occur (Grainger and Holcomb, 2009; Massol et al., 2012).

Grainger and Holcomb (2009) proposed the timing of orthographic, phonological, and semantic processing after reviewing ERP evidence related to the Bimodal Interactive Activation Model (BIAM) of reading. They proposed that for a visually presented word, neural units are activated for sublexical orthographic processing between 100–200 ms, followed by units for othrographic-to-phonological conversion and orthographic word processing between 200–300 ms. These are then followed by units activated for lexical (word) processing between 300–400 ms. Once lexical access has been accomplished, semantic retreival occurs after about 400 ms. Additional ERP research has evidence that orthographic processing of word form might occur as early as 100 ms and that lexical retreival is likely processed later around 250 ms after stimulus presentation (Hauk et al., 2006). In addition, functional connectivity in a visual network revealed sublexical orthographic processing might begin by 85 ms (Herdman, 2011). Thus, the BIAM reading model and timing as proposed by Grainger and Holcomb (2009) appears to indicate that sublexical orthographic and phonological processing should occur by at least 250 ms.

We also should consider the neurophysiological processes involved in learning to read a word when we consider explanations for the timing of phonological retrieval in later skilled reading. By default, learning to read requires a visual stimulus (e.g., letter, bigram, or trigram) to be intimately linked to an auditory stimulus (e.g., phoneme). Over repeated exposures of combined presentations of letters and phonemes, the brain strengthens connections among orthogrpahic and phonological processing units for these stimuli. If we follow the logic of Hebbian plasticity, then viewing letters or words alone by skilled readers should activate these phonlogical processing units. Given that the timing of orthographic processing appears to occur by latest 170 ms for letters and words, we suspect that phonological retrieval must also begin by this time. If phonology and orthography are intimately linked and processed in a parrallel fashion, as we assume above, then rhyming effects that are occuring around 450 ms are far too late in the processing stream to reflect phonological retrieval. Such late rhyme effects more likely reflect susbequent phonological comparisons between stimuli. Not surprisingly, ERP studies using phonological priming have revealed evidence for early (<200 ms) phonological retrieval (e.g., Proverbio et al., 2004; Goslin et al., 2006; Holcomb and Grainger, 2006). In addition, Ashby et al. (2009) revealed syllabic differences in ERPs occurred as early as 80 ms, suggesting that phonetic feature extraction might begin very early in the visual processing stream. Most of these studies used word stimuli and thus a level of lexical processing might also be recruited. In order to evaluate sublexical phonological processing, we investigated the existence of early (<300 ms) ERP differences associated with rhyme processing of single letters.

Another goal of the current study was to evaluate the relative timing of orthographic and phonologic processing for single letters within one study. Previous studies showed ERP difference between orthographic (letters) and non-orthographic (pseudoletters) stimuli occurred around 150–170 ms (Wong et al., 2005; Coch et al., 2008b; Xue et al., 2008; Herdman and Takai, 2013; Stevens et al., 2013). Researchers have suggested that this interval involves an initial stage of orthographic processing (Xue et al., 2008; Herdman, 2011; Massol et al., 2012; Herdman and Takai, 2013). As introduced above, reading models (e.g., BIAM) indicate that phonological processing occurs subsequent to orthographic processing or overlapping this interval (McCandliss et al., 2003; Grainger and Holcomb, 2009; Massol et al., 2012). Because this later supposition is based on evidence across studies, we investigated this possibility using a repeated-measures study design of comparing results among letter-detection and letter-rhyme tasks.

To accomplish the aforementioned goals, we investigated the timing of ERP differences during two letter-rhyming tasks to evaluate the timing of sublexical phonoligcal processing and a letter-identification task to evaluate the timing of sublexical orthographic processing. We obtained evidence for early (<300 ms) and late (>300 ms) ERP differences between rhyming and non-rhyming stimuli. In addition, we obtained evidence from ERP differences for the timing of the early stages of orthographic and phonological processing (around 200 ms).

## Materials and Methods

### Participants

Eighteen adults between the ages 20 and 35 participated in this study (9 female, 9 male). Fourteen participants were right handed, two participants were left handed, and two participants had no preference. English was the primary language for all participants, and all were literate. All participants had normal or corrected to normal (glasses or contacts) vision as tested with a standard Snellen chart. There were no participants with self-reported sensory impairments, cognitive challenges/impairments, or mind-altering drugs/medications. Two participants’ data were excluded due to poor EEG signal-to-noise ratios after artifact rejection and one participant’s data were excluded due to large stimulus-locked alpha and poor behavioral responses. Thus, 15 participants’ data were included in the final analyses. All volunteers were paid $10/h for their participation. Ethics was approved by the Behavioral Research Ethics Board at the University of British Columbia (#H11-01652).

### Stimuli

Visual stimuli were white letter and pseudoletter characters that were individually presented in the center of a black background. Letter stimuli were 12 uppercase letters: A, B, D, E, G, H, J, N, P, R, T, and U. Pseudoletter stimuli were created by segmenting and rearranging the line forms of the letter stimuli in order to reduce the differences in the physical properties between letter and pseudoletter stimuli. The letter stimuli were used for all scenario blocks (LetterID, Paired-Rhyme, and Letter-Rhyme) as described below. Pseudoletter stimuli were only presented in the LetterID block. All stimuli were presented on a 19-inch LCD monitor (DELL/1908FPC) set at a distance of approximately 65 cm from the participant’s eyes. A single character covered about 2–3° of vertical and horizontal visual angle. A white dot at the center of the screen appeared before and after each stimulus for all three scenarios to serve as a visual fixation point.

### Procedures

Procedures were explained to participants prior to beginning the session, and any questions or concerns were addressed before the consent form was signed. The study consisted of three task blocks, presented in the following order: LetterID, Paired-Rhyme, and Letter-Rhyme. This order of task was maintained for all participants in order to minimize a potential confound of participants being primed to perform phonological retrieval during the LetterID task if they previously performed either the Letter-Rhyme or Paired-Rhyme tasks. The Paired-Rhyme task always preceded the Letter-Rhyme task to minimize the possible confound of participants being primed to judge whether or not any displayed letter (first or second presentations) rhymes with a target sound (in this study it was the sound /i:/).

The LetterID task was a two-forced choice experiment whereby a participant was asked to identify the stimulus as a letter or a pseudoletter by pressing one of two corresponding keyboard buttons as fast and as accurately as possible. Each stimulus trial consisted of displaying a stimulus (Letter or Pseudoletter) for a duration of 500 ms followed by a white fixation dot for a randomized duration between 1250–1750 ms. A total of 288 letter and 288 pseudoletter stimuli were displayed over two blocks with a 30–60 s break between blocks. Every sixth trial was designated as a blink trial to encourage participants not to blink during the stimulus trials. A blink trial consisted of presenting the word “Blink” in white text for 1000 ms followed by a fixation dot for 500 ms. Total presentation time for both LetterID blocks was approximately 1380 s (23 min).

The Paired-Rhyme task was a two-forced choice, paired-stimulus experiment whereby a participant was asked to determine (by pressing one of two buttons) whether the letter name of the second stimulus rhymed with the letter name of the first stimulus. The letters in each pair were selected to have 50% rhyming (from set {A, J} and set {D, E, G, P, T, B}) and 50% nonrhyming pairs (randomly selected from set {A, B, D, E, G, H, J, N, P, R, T, and U}). Forty rhyming and 40 nonrhyming pairs were randomly presented in a block. Two blocks of the Paired-Rhyme task were presented to the participant with a 30–90 s break between blocks. Each Paired-Rhyme stimulus trial was presented as follows: the first letter (LetterS1) of the pair was displayed for 500 ms, then a fixation dot was displayed for 750 ms, then the second letter (RhymeS2 or NonRhymeS2) of the pair was displayed for 500 ms, and then a white fixation dot was displayed for a randomized duration between 1250–1750 ms prior to the next paired-stimulus trial. Every sixth trial was designated as a blink trial (trial timing as described above) to encourage participants not to blink during the stimulus trials. Participants were then asked to press a “yes” button whether the pair rhymed and a “no” button whether the pair did not rhyme. Total presentation time for both Paired-Rhyme blocks was approximately 620 s (10.3 min).

The Letter-Rhyme task was a two-forced-choice experiment whereby a participant was asked to press one of two buttons corresponding to whether the name of a letter stimulus rhymed with the sound /i:/ (as in the word “bee”) or did not rhyme with the sound /i:/. For this task only the letter stimuli (set {A, B, D, E, G, H, J, N, P, R, T, and U}) were pseudorandomly displayed so that 50% of the stimulus trials had letter names that rhymed with the sound /i:/ (Rhyme set {B, D, E, G, P, and T}) and 50% of trials had letter names that did not rhyme with the sound /i:/ (NonRhyme set {A, H, J, N, R, and U}). A total of 144 Rhyme and 144 NonRhyme letters were presented. The timing of the stimulus trials were identical to the LetterID task (see above), including a blink trial at every sixth trial.

Throughout all tasks, participants were asked to make button presses as accurately and as fast as possible. Button press were made on a USB keyboard connected directly to the stimulus computer which monitored and recorded the keyboard activity time locked to the stimulus onset. Button press codes and timing were also sent to the BIOSEMI recording system along with the stimulus onset timing and codes. Participants were asked to minimize body and eye movements unless the blink signal was shown. The participants were encouraged to communicate with the experimenter between blocks if they experienced fatigue and needed a break or had any questions. The duration of the entire experiment (including informed consent, eye screening, and EEG setup) was about 2 h.

### Electrophysiological Recording and Analyses

Participants were seated in a comfortable chair located in a sound attenuated-booth. EEG signals were continuously recorded using an ActiView2 64-channel system (BioSemi, Netherlands). The 64 channels were arranged in an expanded 10–20 system using electrode caps suitable for each participant’s head size as determined by head circumference and measuring the nasion, inion, Fz, Pz, T8, and T9 electrode-cap positions. The 64 scalp channels were referenced to a common electrode placed between CPz and CP2; and later referenced to linked mastoid electrodes for offline analyses. Additional bipolar electrodes were placed near the right and left outer canthi (horizontal electrooculography) and infra- and supra-orbital margins (vertical electrooculography) to record and aid in identification of eye movements and blinks. EEG signals were amplified and sampled at a rate 1024 Hz with a band-pass filter of 0.16–208 Hz.

ERPs of −500 to 1000 ms were time locked to the onset of the stimuli in LetterID block (yielding Letter and Pseudoletter trials) and to the onset of stimuli in the Letter-Rhyme (yielding Rhyme and NonRhyme trials). For the Paired-Rhyme block, ERPs of −500 to 1000 ms were time locked to the first stimulus (yielding LetterS1 trials) and to the second stimulus (yielding RhymeS2 and NonRhymeS2 trials). Only stimulus trials with correct button presses to the corresponding stimuli (i.e., Hits) were included in the ERP analyses. Trials with ERPs exceeding ±100 microV between −350 to 850 ms were rejected from further analyses. We subsequently performed a principal component artifact reduction procedure with a principal component threshold of ±100 microV between −1000 to 2000 ms in order to reduce the rising and falling edges of artifacts that might remain within the interval of −350 to 850 ms window (Picton et al., 2000). This ensured that the artifacts did not contaminate the prestimulus interval during baseline correction between −200 to 0 ms. Artifact free trials were then down sampled to 512 Hz, averaged across stimulus trials (as defined above), and filtered using a 30-Hz low-pass filter to obtain evoked potentials (EPs) for each stimulus type. Difference EPs were also calculated for *a priori* defined contrasts Letter minus Pseudoletter, Nonrhyme minus Rhyme, NonRhymeS2 minus RhymeS2, Nonrhyme minus Rhyme stimuli averaged across the Letter-Rhyme and Paired-Rhyme blocks, and Letter minus Rhyme stimuli averaged across the two rhyme task blocks. For each contrast, we performed statistical testing on EP differences across 359 samples between −100 to 600 ms using Student *t*-tests at each scalp electrode. Statistical results were corrected at a false-discovery rate with initial alpha-levels of 0.05 and 0.01 for the 359 samples (Benjamini and Hochberg, 1995). On reflection of our study, we performed a *post hoc* analyses of different EPs in the Letter-Rhyme task that attempted to determine whether or not participants learned to group and identify the rhyme and nonrhyme stimuli strictly on a visual basis and not with respect to their letter name sounds (i.e., phonemic features). The same statistical procedures were followed as described above.

We also calculated the global field power (GFP) for each participant by averaging the EPs (defined above) across all scalp channels, excluding electrooculographic channels. We performed statistical analyses on the GFP waveforms using the same contrasts and statistical procedures as defined for the EPs above.

### Behavioral Recording and Analyses

Participants’ accuracies and reaction times were measured from their button presses for each task. All tasks were a two-force-choice experiment, thus button presses were classified as correct responses (button code matched stimulus type), incorrect responses (button code did not match stimulus type), or missed (no button press). Reaction times for correct, incorrect, and missed responses were measured as the difference in timing between the button codes and the stimulus onset. Only trials that had reaction times between 100–1500 ms were included. This was done to remove inadvertent button presses and extremely delayed button presses that might have resulted from distraction or cognitive fatigue. Reaction times were averaged across trials for each participant. One-way ANOVAs were performed on accuracy and reaction times for the Letter-Rhyme and Paired-Rhyme tasks. Tukey’s *post hoc* analyses were performed on significant ANOVA results. Student *t-tests* were performed on accuracy and reaction times for the LetterID task. Statistical results were considered significant at *p* < 0.05.

## Results

### Behavioral Results

For the LetterID task, participants were similarly accurate at identifying a stimulus as a letter or a pseudoletter (see Table 1 for means and standard deviations). We found no statistical evidence of a difference in accuracy between identifying the letter or pseudoletter stimuli (*t* = −0.19; df = 14; *p* = 0.852). However, we found statistical evidence (*t* = −3.09; df = 14; *p* = 0.008) that reaction times were faster by 18 ± 22 ms to letters than to pseudoletters (Table 1).

**Table 1:** **Behavioral results**.

Task/Condition	Accuracy (%)	Reaction time (ms)
**LetterID**
Letter	98 ± 1	495 ± 42
Pseudoletter	98 ± 1	513 ± 42
**Letter-Rhyme**
Rhyme	93 ± 8	590 ± 71
NonRhyme	90 ± 9	617 ± 61
**Paired-Rhyme**
RhymeS2	78 ± 11	851 ± 86
NonRhymeS2	83 ± 11	848 ± 81

Comparing between rhyming tasks (Letter-Rhyme and Paired-Rhyme), participants were more accurate at identifying stimuli, averaged across rhyme and nonrhyming stimuli, during the Letter-Rhyme than Paired-Rhyme task (Table 1). ANOVA results showed this main effect of task to be significant (*F* = 15.75; df = 1,14; *p* < 0.001). Participants’ accuracy at identifying rhyming and nonrhyming stimuli, as averaged across rhyming tasks, were not statistically different (*F* = 0.93; df = 1,14; *p* = 0.351). We did; however, find statistical evidence for a significant interaction between task and stimulus type (*F* = 15.75; df = 1,14; *p* < 0.001; Table 1). *Post hoc* analyses of this interaction were consistent with the main effects that Rhyme vs. NonRhyme and RhymeS2 vs. NonRhymeS2 were not significantly different (*p* > 0.05). The significant interactions (*p* < 0.05) were Rhyme vs. RhymeS2, Rhyme vs. NonRhymeS2, and NonRhyme vs. RhymeS2.

Reaction times, averaged across stimulus type (rhyme and nonrhyme), were significantly faster by 246 ± 71 ms during the Letter-Rhyme than Paired-Rhyme task (*F* = 186.99; df = 1,14; *p* < 0.001). Reaction times for rhyming and nonrhyming stimuli, average across rhyming task (Letter-Rhyme and Paired-Rhyme), were similar and ANOVA results revealed no evidence for a statistical difference (*F* = 2.65; df = 1,14; *p* = 0.126). ANOVA results revealed a significant interaction between task and stimulus type (*F* = 9.96; df = 1,14; *p* = 0.007). *Post hoc* analyses of this interaction revealed that all mean comparisons were significantly different except for RhymeS2 vs. NonRhymeS2 (*p* < 0.05).

### Electrophysiological Results

#### Letter-Pseudoletter Effects

**Figure 1:**
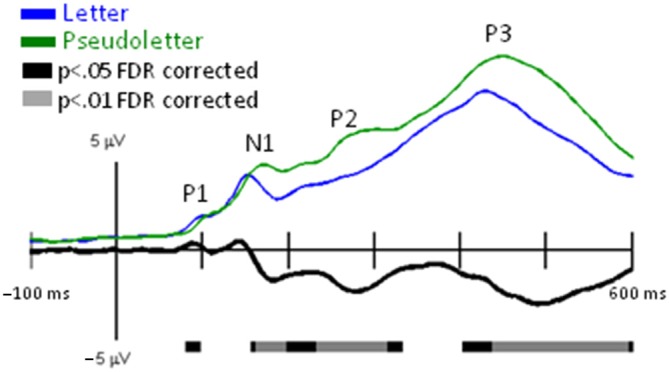
**Global Field Power (GFP) for Letter vs. Pseudoletter Effects.** Grand-mean GFP time-locked to Letter (blue line), Pseudoletter (green line) stimuli, and Letter minus Pseudoletter (black line). Bars below the waveforms designate intervals of significant differences between Letter and Pseudoletter conditions at FDR corrected levels of *p* < 0.05 (black bars) and *p* < 0.01 (gray bars). Peaks within the evoked-potentials (EPs) are labeled above their likely corresponding peaks in the GFPs.

Grand-mean GFPs for Letter and Pseudoletter stimuli showed peaks that corresponded to the typical visual-related (P1, N1, and P2) and attention-related (P3) EPs (Figure 1). Significant differences between GFPs for letters and pseudoletters occurred as early as 85 ms (rising edge of P1) and spanned out to at least 600 ms (end of our analysis interval). The main difference was that GFP to pseudoletters was greater than to letters from about 160–600 ms. The timing of the GFP revealed a possible earlier rise of the P1 and N1 EPs to letters than pseudoletters, which is also evident in the grand-mean EPs recorded at specific electrode sites as described below.

**Figure 2:**
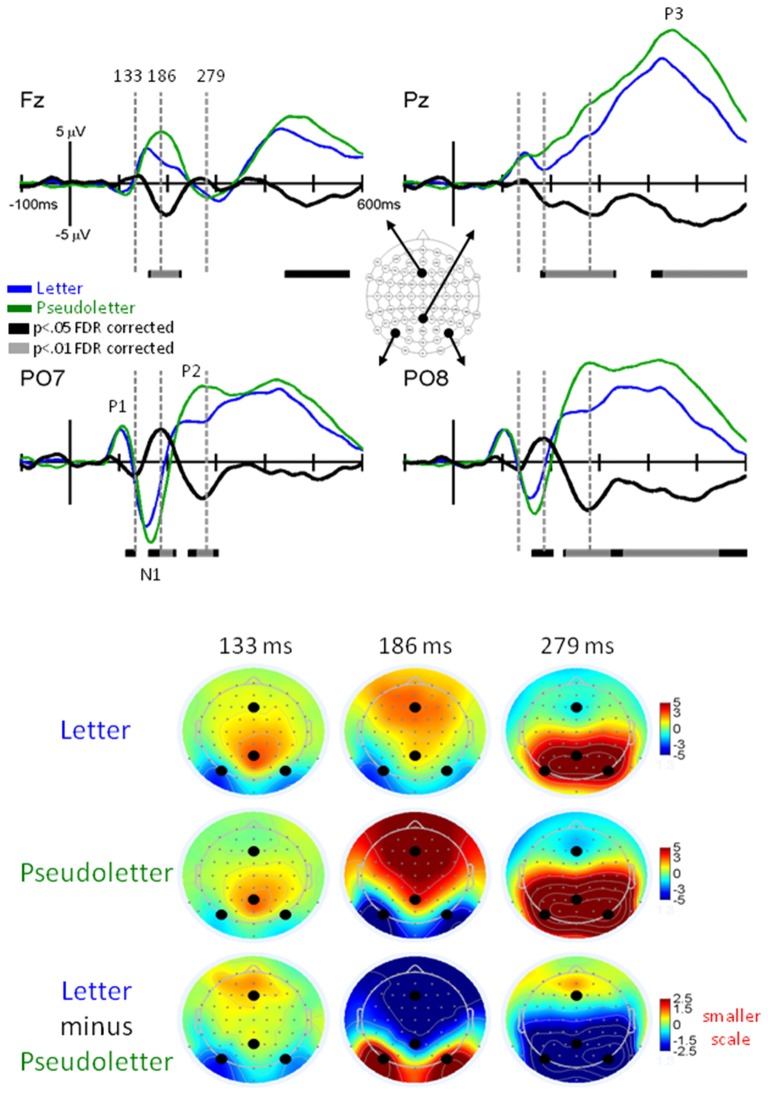
**EPs for Letter vs. Pseudoletter Effects.** Grand-mean EPs to Letter (blue lines), Pseudoletter (green lines), and Letter minus Pseudoletter (black lines) at electrodes Fz, Pz, PO7, and PO8. Bars below the waveforms designate intervals of significant differences between Letter and Pseudoletter conditions at FDR corrected levels of *p* < 0.05 (black bars) and *p* < 0.01 (gray bars). Vertical hash lines designate the latencies for the topographies shown below. The larger black dots in the topographies designate the Fz, Pz, PO7, and PO8 electrode locations. Note the scale for the difference topography (Letter minus Pseudoletter) is half the scale for the Letter and Pseudoletter topographies.

Grand-mean EPs showed typical P1, N1, P2, and P3 waveform morphologies to letter and pseudoletter stimuli (Figure 2). Topographies were also typical for these components showing a parietal-occipital distribution for the P1, N1, and P2 responses and a central-parietal distribution for the P3 response. We found significant differences in EPs to letters and pseudoletters in four time intervals: surrounding the P1 (120–128 ms), N1 (168–210), P2 (250–315 ms), and P3 (350–600 ms) responses. Because the response difference surrounding the P3 was a prolonged effect and had a parietal-occipital topography that was distinct from the P3 topography, we instead classified this difference as a Late Letter-Pseudoletter effect. We note here that using the designations of P1, N1, P2 effects are simply for describing typical EP intervals but we do not assume or imply that the underlying neural generators of the P1 component are necessarily involved in such effects. Additional neural activity can overlap these components. Thus, EP differences might reflect modulation of the P1, N1, and P2 components or they might reflect additional neural activity.

##### P1 Letter-Pseudoletter Effect

EPs to pseudoletter stimuli had delayed P1 responses as compared to EPs to letter stimuli, particularly at electrodes PO7 and PO8 (Figure 2). This difference in timing lead to an apparent EP amplitude difference (pseudoletter > letter) seen in the difference waveforms between 120 and 135 ms (*p* < 0.05 FDR corrected). The topography at 133 ms revealed this effect to be distributed mainly over parietal and occipital scalp regions (Figure 2).

##### N1 Letter-Psuedoletter Effect

EPs to pseudoletter stimuli had greater and delayed N1 responses as compared to EPs to letter stimuli; particularly at electrodes PO7 and PO8 (Figure 2). Difference waveforms (letter minus pseudoletter) clearly showed this effect was significant between 185–210 ms (*p* < 0.01 FDR corrected). The amplitude difference peaked at 186 ms and was mainly distributed over parietal-occipital scalp regions as a positive ERP difference—pseudoletters had more negative EP than letters (Figure 2). The bilateral posterior positive differences between 185–210 ms had inverse (i.e., negative) differences distributed over frontal scalp regions.

##### P2 Letter-Pseudoletter Effect

EPs to pseudoletter stimuli had greater P2 responses than EPs to letter stimuli; particularly at electrodes P7/P8, PO7/PO8, and O1/O2 (Figure 2). Difference waveforms (letter minus pseudoletter) showed that this effect was significant between 240–315 ms (*p* < 0.01 FDR corrected). A peak negative EP difference occurred at 279 ms and was mainly distributed over parietal and occipital scalp regions as shown in the topographies (Figure 2).

##### Late Letter-Pseudoletter Effect

EPs to pseudoletter stimuli had greater positive responses than EPs to letter stimuli; particularly at electrodes P8, PO8, and O2 (Figure 2). Difference waveforms (letter minus pseudoletter) showed that this effect was significant between 350–540 ms (*p* < 0.01 FDR corrected). A peak amplitude difference occurred at approximately 490 ms and was mainly distributed over right parietal and occipital scalp regions.

#### Rhyme Effects

**Figure 3:**
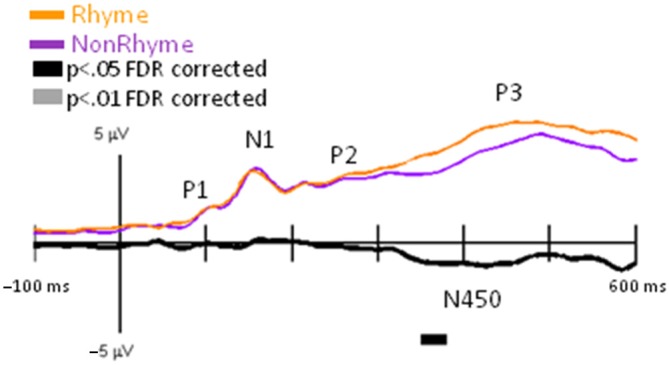
**GFP for NonRhyme vs. Rhyme Effects.** Grand-mean GFP time-locked to Rhyme (orange line), NonRhyme (pruple line) stimuli, and NonRhyme minus Rhyme (black line). Bars below the waveforms designate intervals of significant differences between NonRhyme and Rhyme conditions at FDR corrected levels of *p* < 0.05 (black bars) and *p* < 0.01 (gray bars). Peaks within the EPs are labeled above their likely corresponding peaks in the GFPs.

**Figure 4:**
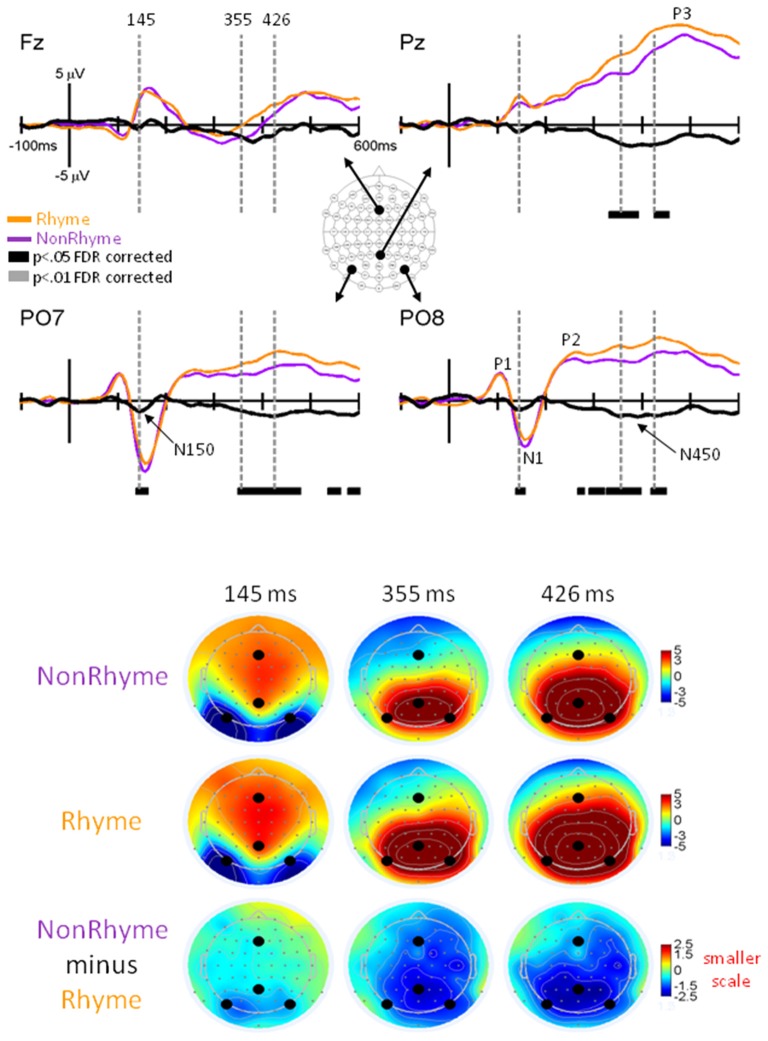
**EPs for NonRhyme vs. Rhyme Effects.** Grand-mean EPs to Rhyme (orange line), NonRhyme (pruple line) stimuli, and NonRhyme minus Rhyme (black line) at electrodes Fz, Pz, PO7, and PO8. Bars below the waveforms designate intervals of significant differences between NonRhyme and Rhyme conditions at FDR corrected levels of *p* < 0.05 (black bars) and *p* < 0.01 (gray bars). Vertical hash lines designate the latencies for the topographies shown below. The larger black dots in the topographies designate the Fz, Pz, PO7, and PO8 electrode locations. Note the scale for the difference topography (NonRhyme minus Rhyme) is half the scale for the Letter and Pseudoletter topographies.

We observed similar timing and morphologies of EP waveforms across the Letter-Rhyme and Paired-Rhyme tasks; therefore, we only presented results for GFP and EP differences between rhyme and nonrhyme stimuli, after they were averaged across these tasks. Grand-mean GFP waveforms were significantly larger between 355–375 ms for nonrhyme than rhyme stimuli (Figure 3). This slow wave difference between these conditions extended to 600 ms, which was likely an N450. This N450 was more pronounced in specific electrode recordings as described below (e.g., Pz, PO7, and PO8 in Figure 4).

Observation of the grand-averaged EPs showed the typical P1, N1, P2, and P3 waveform morphologies to rhyme and nonrhyme stimuli (Figure 4). Topographies were also typical for these components showing a parietal-occipital distribution for the P1, N1, and P2 responses and a central-parietal distribution for the P3 responses. We found two intervals with significant differences (*p* < 0.05 FDR corrected) between rhyme and nonrhyme EPs: an N150 effect between 138–162 ms and an N450 effect between 270–472 ms. In addition, we performed a *post hoc* analyses of EPs in the Letter-Rhyme task that attempted to determine whether or not participants might have shifted to a visual-only letter recognition task later in the block by memorizing the six rhyme and six nonrhyme stimuli as two categories. The statistical analyses revealed that EPs differences from the first- and second-half of the trials were not significantly different (*p* > 0.05). Thus, we found no evidence that EP effects were different between the first and second halves of the Letter-Rhyme block. This indicated that participants likley perfomed the rhyme judgment throughout the Letter-Rhyme block and did not alter their task judgment to a visual-only recognition of the letters in the later part of the block.

##### N150 Rhyme Effect

Comparisons of EPs between rhyme and nonrhyme stimuli displayed a more negative-going wave for the nonrhyme stimuli, particularly at electrodes PO7/PO8 and O1/O2 (Figure 4). Difference waveforms (nonrhyme minus rhyme) revealed this effect to be significant between 139 and 156 ms (*p* < 0.05, FDR corrected). A peak negative difference occurred at 145 ms and was mainly distributed over occipital scalp regions, as displayed in the topographies (Figure 4).

##### N450 Rhyme Effect

EPs to rhyme stimuli were more positive than nonrhyme stimuli starting at about 250 ms and extending to 500 ms. This effect was particularly evident at electrodes P2, PO7/PO8, O1/O2 (Figure 4). Difference waveforms (nonrhyme minus rhyme) confirmed this effect to be significant between 302 and 450 ms (*p* < 0.05, FDR corrected) at electrodes PO7/PO8. This later amplitude difference between nonrhyme and rhyme conditions peaked at 426 ms and was distributed over occipital, parietal, and central regions, as displayed in the topographies (Figure 4). The P3 component also displays a larger frontal-scalp negativity and posterior scalp positivity at 426 ms when observing both the rhyme and nonrhyme waveforms.

#### Letter vs. Rhyme Effects

**Figure 5:**
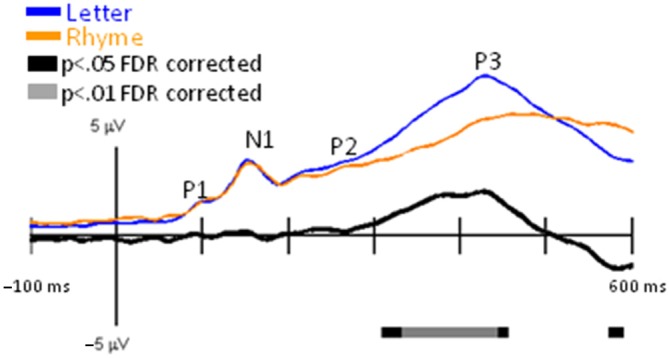
**GFP for Letter vs. Rhyme.** Grand-mean GFP time-locked to Letter (blue line), Rhyme (orange line), and Letter minus Rhyme (black line). Bars below the waveforms designate intervals of significant differences between Letter and Rhyme conditions at FDR corrected levels of *p* < 0.05 (black bars) and *p* < 0.01 (gray bars). Peaks within the EPs are labeled above their likely corresponding peaks in the GFPs.

**Figure 6:**
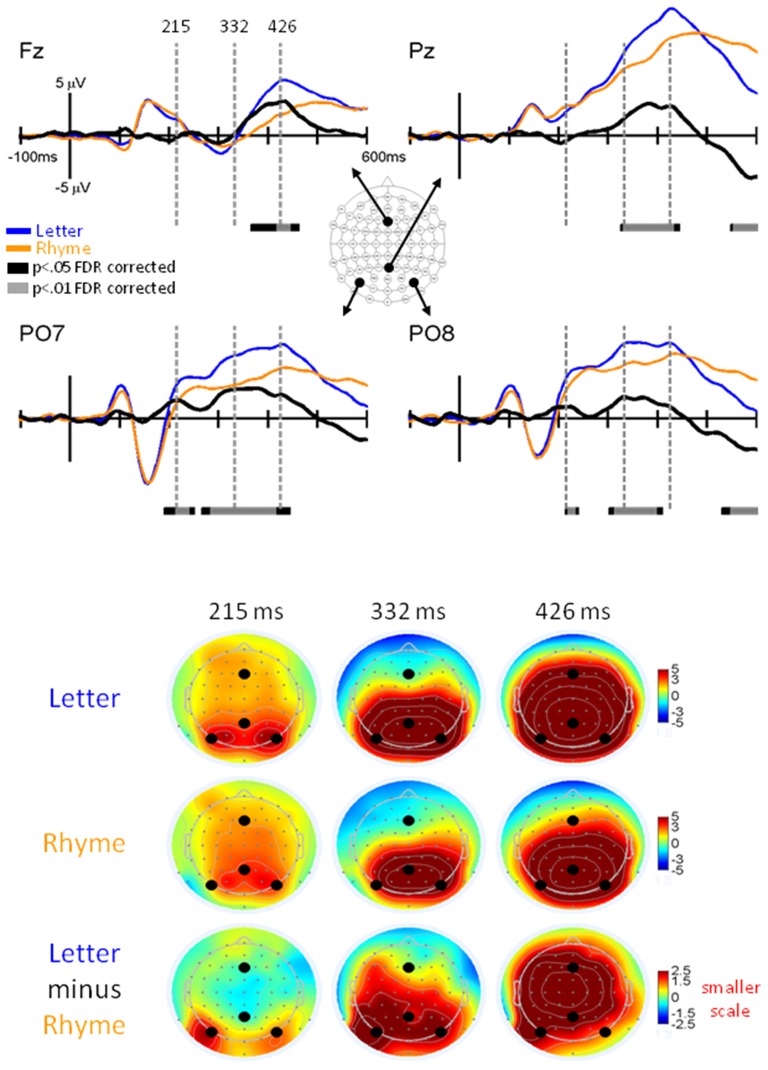
**EPs for Letter vs. Rhyme Effects.** Grand-mean EPs to Letter (blue line), Rhyme (orange line), and Letter minus Rhyme (black line) at electrodes Fz, Pz, PO7, and PO8. Bars below the waveforms designate intervals of significant differences between Letter and Rhyme conditions at FDR corrected levels of *p* < 0.05 (black bars) and *p* < 0.01 (gray bars). Vertical hash lines designate the latencies for the topographies shown below. The larger black dots in the topographies designate the Fz, Pz, PO7, and PO8 electrode locations. Note the scale for the difference topography (Letter minus Rhyme) is half the scale for the Letter and Pseudoletter topographies.

GFPs between Letter and Rhyme conditions revealed significant differences surrounding the P3 interval (Figure 5). This was expected because the Rhyme tasks were more challenging than the Letter-ID task, as revealed by slower reaction times and poorer accuracies for the Rhyme tasks (Table 1). This increase in challenge for the Rhyme tasks caused greater inter-trial and inter-participant variabilities for the P3 and resulted in a delayed and reduced grand-averaged P3 peak for the Rhyme tasks as observed in the GFPs (Figure 5) and EPs (Figure 6).

A main finding of this article was significant EP differences that occurred between 200–245 ms at electrodes PO7 and PO8 (*p* < 0.05 FDR corrected; Figure 6). EPs were larger for Letter than Rhyme conditions in this time interval and the difference waves showed a peak at 215 ms that occurred on the rising edge of the P2 response. In addition to this difference, EPs showed larger and earlier peaking P3 responses at electrode Pz to the Letter than Rhyme conditions (Figure 6). This resulted in apparent amplitude differences between 330–435 ms and 550–600 ms. These P3 effects appeared to be spread over frontal, central, and parietal scalp regions (see lower right topography in Figure 6). From a spatiotemporal perspective, the difference EPs between Letter and Rhyme conditions emerged at 200 ms over bilateral parietal occipital scalp regions which were followed by an overlapping P3 effect between 330–425 ms that was broadly distributed over frontal-central-parietal scalp regions.

## Discussion

Results from the current study provided further evidence for early phonological processing (~150 ms; Holcomb and Grainger, 2006) and early orthographic processing (130–170 ms; Wong et al., 2005; Coch et al., 2008b; Xue et al., 2008; Herdman and Takai, 2013; Stevens et al., 2013), as well as phonological processing at 200–245 ms. This evidence is discussed below with respect to the effects found in the rhyming tasks and the letter identification task.

### Behavioral Effects

In addition to EP differences between letters and pseudoletters, we found behavioral response time differences. Participants pressed a button significantly faster to Letter than to Pseudoletter stimuli by 18 ms. This further replicates previous findings (LaBerge, 1973; Herdman, 2011) and indicates that single letter stimuli are identified faster than pseudoletter stimuli. Adult participants have extensive experience with these familiar letter stimuli and thus form highly-consolidated visual templates. Visual identification of letters are rapid and likely automatic because of this extensive experience. Pseudoletters, on the other hand, are unfamiliar visual objects and require more processing to be able to identify them as non-letters (current study; Herdman, 2011). This delays the information flow to the motor-response-selection networks involved in response execution. These findings indicate that experience with text speeds up neural processing, allowing for a more rapid identification of familiar letter stimuli as compared to unfamiliar pseudoletter stimuli.

### N150 Rhyme Effect

One objective of this study was to determine whether there were early (<300 ms) EP differences between rhyme and nonrhyme stimuli that preceded the commonly reported N450 Rhyme effects (Coch et al., 2008a,b; Stevens et al., 2013). We found early rhyme effects as evidenced by EP differences between rhyme and nonrhyme conditions occurring at around 150 ms. The timing of such an early difference is consistent with ERP studies showing phonological priming to occur around 150 ms (Proverbio et al., 2004; Holcomb and Grainger, 2006). However, our N150 effect displayed a more negative response for the nonrhyme stimuli compared to the rhyme stimuli. The directionality of differences appears to be contrary to previous findings of a more positive ERP to phonologically primed than nonprimed stimuli (Proverbio et al., 2004; and Holcomb and Grainger, 2006). This difference might be attributed to the fact that the previous studies used words instead of letter stimuli (current study), and thus required more lexical retrieval, whereas the letter stimuli in the our study mainly required sublexical processing. Furthermore, these studies involved significantly different tasks compared to the present study. The Holcomb and Grainger (2006) study used prime-target pair task, which gave rise to the N150 effect. Because the prime and target stimuli had either matching (e.g., table-TABLE) or nonmatching (table-MOUSE) word representations from different font-cases, Holcomb and Grainger (2006) interpreted their N150 effect to reflect phonological priming because orthographic features (lower-case vs. upper case) were not primed. This is a logical supposition when considering early visual processing levels. However, priming could occur at the level of the abstract letter unit (ALU), likely akin to the location-invariant orthographic unit (Grainger and Holcomb, 2009), which would presumably consist of orthographic and phonologic representations. It would be difficult to tease out orthographic from phonologic priming using such a paradigm. The Paired-Rhyme task in our study; however, might help shed some light on this issue (with the caveat that our study was a paired-stimulus decision experiment and not a traditional priming experiment). The first letter name (Stim1) is retrieved and stored in short-term memory and then compared to letter name of the following stimulus (Stim2). If Stim1 and Stim2 have letter names that rhyme, the participant judges them as rhymed. What is relevant to this discussion is that Stim1 stimuli are completely dissimilar in orthography to Stim2 because they are different ALUs (i.e., different letters). Only the phonology of the letter names have similar features (e.g., /tee/ and /dee/). This suggests the N150 effect may reflect phonological processing. A highly unlikely caveat to our Letter-Rhyme task was that letters had a chance of being repeated, which might have caused orthographic priming. However, given our randomization of stimuli, repeated letters had a very low chance of occurrence; approximately 0.7% of possible events. Importantly, no letters were repeated in the Paired-Rhyme task between Stim1 and Stim2. Thus, we feel that the present study’s N150 effect cannot be explained by priming at the orthographic level.

However, if the N150 effect observed in the present study was truly due to phonological processing, then it would be expected to also occur in our comparison of ERPs to letter vs. rhyme stimuli across the phonological (Paired-Rhyme and Letter-Rhyme) and orthographic (LetterID) tasks (see Figure 6). Because we didn’t find evidence for the N150 effect in this comparison, the N150 effect for the rhyme vs. nonrhyme comparison can be best described as a rhyming effect that occurs due to a differential recruitment and comparison of rhyming and nonrhyming letters. Further investigations must be done to examine the role of phonological processing, if any, on the N150 effect or if the N150 effect is related to the specific tasks performed in this study.

### N450 Rhyme Effect

In addition to our effect at 150 ms, we found the highly reported N450 rhyme effect showing that nonrhyme stimuli have more negative responses (less positive) than rhyme stimuli (Coch et al., 2008a). Thus, our tasks provided a reliable modulation of the EPs to rhyme and nonrhyme stimuli. This rhyme effect is widely hypothesized to reflect primarily phonological processing (Rugg and Barrett, 1987; Penolazzi et al., 2006). However, there is insufficient evidence on this subject thus far to ascertain that the N450 effect is purely phonological. There is also evidence suggesting that the N450 effect might represent orthographic and phonological mapping (Kramer and Donchin, 1987; Rugg and Barrett, 1987; Weber-Fox et al., 2003). Similar rhyme effects have been shown by Stevens et al. (2013) for single letters as an implicit task without the involvement of rhyme judgment, so this effect is unlikely to be due to rhyme/non-rhyme explicit judgment. This study is consistent with the Stevens et al. (2013) article that suggests the N450 is mostly due to phonological processing and largely unrelated to judgment. Because behavioral response times occurred between 600–800 ms, we believe that the N450 represents a late phonological processing stage involved in awareness. This would be consistent with the phonological processing within later stages involving frontal and parietal cortices. Topography of the N450 had a more central-parietal distribution, which could involve such late stages of processing; however, source modeling would be required to better elucidate such conjecture. Future source modeling studies of the N450 effect for single letters are therefore warranted.

### P1 Letter vs. Pseudoletter Effect

The present study replicated previous findings that pseudoletter stimuli evoked more delayed P1 responses than did letter stimuli (Herdman, 2011; Herdman and Takai, 2013). This P1 letter effect could be due to delayed early visual processing of pseudoletters compared to letters. If so, this indicates that letters have a faster neural recruitment than pseudoletters in early visual processing centers of the brain, such as striate and extra-striate cortices. However, stimulus attributes (e.g., spatial frequency) cannot be fully equated between letters and pseudoletters and might contribute to this early P1 effect. Nevertheless, we attempted to maintain similarity of most of the stimulus attributes (e.g., luminence) by rearranging the line forms of the letters to generate pseudoletters. The true cause of this early P1 effect cannot be fully elucidated from the present study’s results and is a potential area for further research. Because we have seen this effect across multiple studies using different stimulus sets and recording devices (EEG and MEG; present study; Herdman, 2011; Herdman and Takai, 2013), we highly suspect that this early P1 effect is related to initial stages of orthographic processing.

### N1 Letter vs. Pseudoletter Effect

A main letter effect was that pseudoletter stimuli evoked larger and more delayed N1 responses than did letter stimuli. This was a further replication of previous results using MEG (Herdman, 2011) and EEG (Appelbaum et al., 2009; Herdman and Takai, 2013). The EP differences in all time intervals from the current study were strikingly similar to those reported in Herdman and Takai (2013). Herdman and Takai (2013) showed that these EP differences were distributed over posterior-occipital scalp regions with their generators localized to bilateral fusiform gyri. Other researchers, however, have reported significantly larger N1 responses to Letters than Pseudoletters (Tarkiainen et al., 1999; Wong et al., 2005; Stevens et al., 2013) or no significant N1 differences between letters and other non-letter control stimuli (geometric forms, faces, etc; Pernet et al., 2003). As such, studies have reported varied results of N1 effects for single-letter processing. A possible explanation for these inconsistent findings might be due to differences among the tasks. In the Stevens et al. (2013) study, participants were asked to perform a 1-back task comparing visual templates between repeated stimulus events. Participants in the current study (and previous Herdman studies) were asked to compare letters and pseudoletters to well-learned (endogenous) alphabetic templates. At this time, we are unable to conclude whether differences in experimental design could explain why our replicated findings of N1 effects (current study; Herdman, 2011; and Herdman and Takai, 2013) are different from those reported previously (Tarkiainen et al., 1999; Wong et al., 2005; Stevens et al., 2013). Future work directly comparing these two type of tasks (1-back vs. identification) for differences in EPs between Letters and Pseudoletters will be required to evaluate whether or not task differences exist. We believe that differences in attentional demands of such tasks are likely not factors because our previous work showed that attention directed to or away from orthography had no significant effect on the early EPs to letters and pseudoletters (Herdman and Takai, 2013).

Furthermore, a major challenge in comparing our findings with previous research is the limited number of other studies providing/displaying difference waves (letter minus pseudoletter) and the way in which amplitude measures were calculated. For example, Stevens et al. (2013) averaged the EP amplitudes across the time samples of ±25 ms surrounding the N1 peak to letter and pseudoletter stimuli and then calculated the difference in this averaged N1 amplitude between letter and pseudoletter conditions. This analysis method assumes that the main difference in EPs to letters compared to pseudoletter occurs at the N1 peak. As has been noted previously in the ERP literature (Handy, 2005), stimuli and task effects can occur as modulations of transient EP components or as additional EP components that overlap transient responses with shifted temporal dynamics. As we have shown in our articles (current study; Herdman, 2011; Herdman and Takai, 2013), the main Letter-Pseudoletter effect appears to be a broadening and/or delay of the N1 response to pseudoletters than to letters (see Figure 1). Thus, we surmise that the main difference is most likely due to additional processing or an additional component that overlaps the N1 response. By displaying and analyzing the difference waveforms, we were able to identify that the significant processing differences between letters and pseudoletters occurred between 185–210 ms. The peak of the difference occurs slightly later than the peak of the N1. This is most likely an apparent amplitude difference created by the delayed processing of pseudoletters. Thus, we believe that the findings of the present study regarding the N1 effect are consistent with the idea that pseudoletter stimuli require greater and more prolonged processing than letter stimuli.

### P2 Letter vs. Pseudoletter Effect

Unlike the N1 effect, the P2 effect showing a larger positivity to pseudoletters than letters is consistent across most studies (Wong et al., 2005; Appelbaum et al., 2009; Herdman, 2011; Herdman and Takai, 2013; Stevens et al., 2013). Interestingly, topographical distribution of the P2 effect was more right-hemispheric dominant. This result is also consistent with previous studies, which showed that this P2 effect was localized predominantly to the right inferior temporal gyrus (Herdman and Takai, 2013). This P2 effect supports the idea that unfamiliar objects require more processing for identification and categorization (Appelbaum et al., 2009; Herdman, 2011; Herdman and Takai, 2013). Such an interpretation is consistent with previous theoretical models of letter/pseudoletter processing, which state that neural templates (i.e., ALUs) exist to allow for more efficient processing of familiar letters (McClelland and Rumelhart, 1981; Price, 2000; Grainger and Holcomb, 2009). However, uncertainty still exists in whether this effect is due to purely orthographic processing or parallel orthographic and phonological processing.

### Late-Letter Effect

The late-letter effect occurred as a greater positivity to pseudoletter stimuli than to letter stimuli that was mostly right-hemispheric dominant. This effect was previously shown but not discussed in our previous report (Herdman and Takai, 2013). This late letter effect may be evidence for more prolonged processing of pseudoletters compared to letters, consistent with the results for the earlier effects found by the present study. The interval of this effect (350–540 ms) occurred around the average behavioral response time (495 ms) and was fairly late in the processing stages. Thus, this EP difference could reflect greater feedback from response selection processes to visual centers in an attempt to consolidate pseudoletter templates for improving performance on subsequent events. Feedback for Letter identification would be less because there would be less need to consolidate highly-familiar letter templates.

### Letter vs. Rhyme Effects

The P2 effect around 200–280 ms observed for the letter vs. rhyme stimuli comparison can be best explained by differences in phonological processing. The need for phonological processing can be assumed for the rhyme tasks (Paired-Rhyme and Letter-Rhyme), whereas the LetterID task can be performed with orthographic/nonorthographic distinctions as early as 130 ms (see above). The rhyme tasks then require decisions based on further sublexical phonological processing of the stimuli. Thus, the activity represented by the P2 effect is likely due to sublexical phonological processing of the rhyme stimuli as opposed to lesser such processing for letter stimuli in the letter vs. pseudoletter task. This is supported by the fact that the P2 effect was not observed in comparing rhyme vs. nonrhyme stimuli, suggesting that sublexical phonological processing was also required for nonrhyme stimuli, as expected by the task requirements. A caveat to this hypothesis is that the orthographic processing required for the letter stimuli in the letter vs. pseudoletter task cannot be viewed as distinct and separate from phonological processing. However, even with the assumption that some phonological processing may have occurred for letter stimuli, the P2 effect observed between these two tasks as a result of differences in sublexical phonological processing is well supported by past studies (Bentin et al., 1999; Proverbio et al., 2004; Simon et al., 2006). In a study by Bentin et al. (1999) using a rhyme decision task with words, pseudowords, and nonwords, an N320 effect was observed between pronounceable (word and pseudoword) and nonpronounceable (nonword) stimuli. This effect was hypothesized to be due to phonological effects, between orthography and phonology. In the context of word stimuli, this N320 effect represents sublexical grapheme-to-phoneme conversion (Bentin et al., 1999; Proverbio et al., 2004). The majority of such studies involving rhyme decision tasks used word stimuli instead of letter stimuli, which could be a reason for the faster onset of the P2 effect observed in our study, as the phonology of single-letter stimuli are likely accessed quicker than whole-word stimuli. Interestingly, the N320 effect observed by Bentin et al. (1999) had a slightly more left occipital-parietal distribution whereas the P2 effect observed in this study had a bilateral occipital-parietal scalp distribution.

The main findings from this study support two conclusions. First, differences in neural processing of single letters vs. pseudoletters between 130–190 ms revealed that letters are processed earlier and possibly faster within the brain than pseudoletters. This likely resulted in the observed 18 ms faster behavioral reaction times for letters than pseudoletters. Second, results from the tasks showed that early neural processing differences (150–200 ms) between letter and rhyme stimuli likely reflect sublexical phonological processing. Taken together, the main findings from our study fill in evidence for the temporal dynamics of orthographic and phonological processing of single letters that are consistent with the temporal dynamics presented in reading models (Grainger et al., 2003; McCandliss et al., 2003; Holcomb and Grainger, 2006; Grainger and Holcomb, 2009; Massol et al., 2012).

## Author Contributions

SAB is the primary author. SAB collected, analyzed, and wrote major portions of the manuscript. ATH is the senior author. ATH designed, analyzed, and wrote major portions of the manuscript.

## Conflict of Interest Statement

The authors declare that the research was conducted in the absence of any commercial or financial relationships that could be construed as a potential conflict of interest.
